# Analysis of Factors Influencing the Willingness of Chinese Older Adults to Use mHealth Devices: Nationwide Cross-Sectional Survey Study

**DOI:** 10.2196/66804

**Published:** 2025-03-04

**Authors:** Mengyao Yan, Wendi Sun, Cheng Tan, Yibo Wu, Yuanli Liu

**Affiliations:** 1 Chinese Academy of Medical Sciences & Peking Union Medical College Beijing China; 2 School of Humanities and Management Zhejiang Chinese Medical University Zhejiang China; 3 School of Government Peking University Beijing China; 4 School of Public Health Peking University Beijing China

**Keywords:** older adults, mobile health devices, health management, medical services, mobile phone

## Abstract

**Background:**

In addition to standard older adult care services, mobile medical devices have proved to be an effective tool for controlling the health of older adults. However, little is known about the variables driving the acceptance of these gadgets and the willingness of older adults in China to use them.

**Objective:**

This study aims to explore the factors that affect the use of mobile health (mHealth) devices by older adults in China, focusing on individual, social, and family influences.

**Methods:**

The Psychology and Behavior Investigation of Chinese Residents survey database provided the data for this study. The survey was conducted in 148 Chinese cities between June 20 and August 31, 2022. The parameters linked to older persons’ desire to use mobile medical devices were determined by this study using a combination model of multiple stepwise linear regression and a classification and regression tree decision tree.

**Results:**

In total, 4085 older adults took part in the poll. On a scale of 0 to 100, the average score for willingness to adopt mHealth devices was 63.70 (SD 25.11). The results of the multiple stepwise linear regression showed that having a postgraduate degree and higher (β=.040; *P*=.007), being unemployed (β=.037; *P*=.02), having a high social status (β=.085; *P*<.001), possessing high health literacy (β=.089; *P*<.001), demonstrating high self-efficacy (β=.043; *P*=.02), not living with children (β=.0340; *P*=.02), having a household per capita monthly income of >Y4000 (US $550) (β=.048; *P*=.002), experiencing high perceived social support (β=.096; *P*<.001), reporting a high quality of life (β=.149; *P*<.001), having higher levels of family communication (β=–.071; *P*<.001), having an identity bubble (β=.085; *P*<.001), not having chronic diseases (β=.049; *P*=.001), and experiencing mild depression (β=–.035; *P*=.02) were associated with older adults’ willingness to use mHealth devices. The classification and regression tree decision tree model’s findings demonstrated that the primary determinants of older adults’ desire to use mHealth devices are quality of life, identity bubble, social status, health literacy, family health, and perceived social support.

**Conclusions:**

This study uses the Andersen Healthcare Utilization Model to investigate the effects of demand variables, enabling resources, and predisposing traits on older persons’ propensity to use mHealth devices. These results offer reference data for the marketing and use of mHealth devices for older individuals in the future. The ultimate goal of this strategy is to create a balanced and harmonious integration of technology and humanistic care.

## Introduction

### Background

In the context of the rapidly advancing global demographic shift toward aging and the exponential growth of mobile health (mHealth) technology, mHealth devices have emerged as pivotal instruments for augmenting the quality of life of older adults, fostering proactive health self-management, and mitigating the effects of aging. As the nation harbors the world’s largest older adult population, China confronts the imperative of effectively harnessing mHealth technology to cater to the extensive needs of its aging populace. According to statistics from the National Bureau of Statistics of China from December 2023 [1], China was home to 296.97 million individuals aged ≥60 years, constituting 21.1% of the national population, with 216.76 million of these being aged ≥65 years, representing 15.4%. This pervasive aging trend is concurrent with a surge in the incidence of chronic diseases [2], as more than two-thirds of Chinese older adults aged ≥65 years contend with multiple comorbidities. However, in 2022, China recorded a physician-to-population ratio of 3.15 practicing (assistant) physicians per 1000 individuals, alongside a ratio of 3.71 registered nurses per 1000 population—figures that, while placing the country in the middle tier internationally [3], nonetheless present a formidable challenge to the effective management of chronic conditions and the provision of comprehensive public health services. These circumstances significantly strain the nation’s capacity to manage chronic illnesses and deliver robust public health services.

The World Health Organization’s Draft Global Strategy on digital health 2020 to 2025 underscores the strategic importance of digital health within global health care systems. The Chinese government actively promotes an “internet+medical service” development model, vigorously driving forward initiatives for intelligent health and older adult care services. The clinical practice of the 2023 hypertension guidelines update has been released, for the first time incorporating wearable devices into its recommendations, presenting them as a novel modality for blood pressure measurement, working in conjunction with traditional methods, such as home blood pressure monitoring, to fortify the health defense against hypertension. mHealth, as a novel paradigm facilitating the exchange and management of health care information via mobile devices, assumes a crucial role in health management, demonstrating immense potential in domains such as chronic disease management, fall prevention [4], activity monitoring [5], and individual health behavior tracking [6]. It offers personalized and precision health care services [7], enabling the creation of individualized health profiles through integration with hospital systems. In turn, it assists in alleviating issues related to the unequal distribution of medical resources and health care services [8]. An example is the HUAWEI Watch D, which, having secured class II medical device registration certification from national authorities and undergone international standard validations, garners endorsement.

Moreover, against the backdrop of increasing empty-nest syndrome among the older adult population and the integration of medical and older adult care services in China, mHealth devices can be synergistically combined with health care services to enhance the quality and efficiency of older adult care provision. Data from the 2022 National Report on the development of national undertakings for the older adults by the National Bureau of Statistics of China reveal that, as of the end of 2022, China boasted 387,000 older adult care institutions and facilities nationwide, housing 8.294 million beds dedicated to older adult care services. Even with a conservative estimate adhering to the national standard ratio of 1 caregiver per 4 older adults, there is a pressing need for at least 2.075 million caregivers. However, the current workforce comprises only approximately 300,000 caregivers, leaving a substantial shortfall [9]. mHealth devices can, to a certain extent, mitigate this shortage of personnel. For instance, Apple’s iOS 16 and watchOS 9 now come bundled with a medication reminder app within their native “Health” software, assisting older adults with medication adherence. In Beijing’s Xicheng district, authorities have installed smart caregiving mattresses in older adult homes, continuously monitoring vital signs, such as heart rate and respiratory patterns, as well as bed entry and exit times, thereby augmenting care provision without direct human intervention.

In the field of commercial layout, the market size of smart wearable devices is expanding and will still maintain rapid growth in the next few years. International Data Corporation’s latest quarterly tracking report on China’s wearable devices market shows that China’s wearable devices market shipped 34.7 million units in the third quarter of 2023, a year-on-year increase of 7.5%, and the overall market continues to grow. International Data Corporation expects that in 2024, the number of adult smartwatches will increase by 11%, driven by diversified product offerings [10].

Despite the multitude of benefits associated with mHealth, several critical challenges persist in its practical implementation. Previous studies have examined the willingness of the general Chinese population to use mHealth devices [11]; however, given the differences in digital divide, health literacy, technology acceptance, social support network, and concept of life among the older adults, the application of mHealth devices in the older adults has not reached an ideal state. Particularly in China, studies have shown that the older adults and the groups that are chronically ill, who are the potential main users, are not very receptive to mHealth services [8]. Furthermore, concerns over data security and accuracy [12], affordability of devices and associated services, equitable access [13], insufficient age-appropriateness of the products, anxiety induced by data-driven insights, and marginalization of certain functionalities, such as fall detection, all contribute to a less-than-ideal adoption scenario. Therefore, the willingness of the older adult population to use mHealth devices remains a larger issue.

In summary, while mHealth devices are being developed for a wider range of functions and see significant growth on the supply side, demand-side research primarily focuses on factors influencing the willingness to use these devices. These factors include perceived usefulness, product innovation, privacy protection, self-efficacy, self-perceived aging, and health awareness among users [14-17], and there is a significant lack of a large-scale sample of the older adult population. Therefore, in this dissertation, a large-scale cross-sectional survey was conducted nationwide with the aim of revealing the current status of Chinese older adults’ willingness to use mHealth devices and the key influencing factors behind it. This is of great significance for the global understanding of the characteristics of the acceptance of new technologies among the older adult population, enhancing the effectiveness of digital health interventions and promoting the development of the smart aging industry, as well as providing a valuable empirical basis for international health equity and policy making for an aging society.

### Theories and Hypotheses

**Figure 1:**
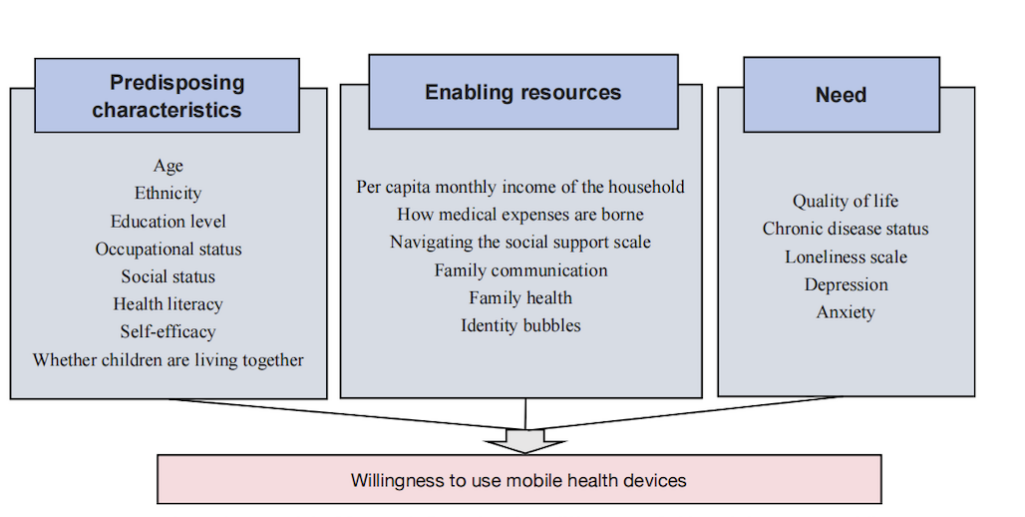
A model of factors influencing the willingness of older adults to use mobile health devices.

The Anderson Behavioral Model of Health Services Use is the model of choice in the field of health services to explain and predict health care behaviors as well as to analyze the factors influencing health services use behaviors of different households. The model identifies predisposing characteristics, enabling resources, and need as key factors affecting the use of health services [18]. This contributes to the systematic analysis of factors influencing individual health service use behavior and the evaluation of the accessibility of health service use [19]. From the viewpoint of the population to which the study applies, the Anderson model is applicable to the study of the health care service use behavior of the general population as well as the study of the health care service use behavior of special populations, such as older adults, women, low-income earners, children, and people with the HIV infection. In terms of the scope of application, the model is applicable to the study of the entire process of individual health service use behavior, including the study of factors influencing the choice of individual health care modality, medical costs, disease screening, drug use, and so on [20,21]. This study views mHealth device use as a form of health service delivery and therefore uses the Anderson Health Service Utilization Model to explore the impact of different factors on mHealth device use. The specific model is shown in Figure 1.

On the basis of this hypothesis, the following propositions are made, informed by theory and existing research:

- Hypothesis 1: older adults will be more likely to be willing to use mHealth devices than non–older adults.
- Hypothesis 2: older adults’ willingness to use mobile devices is positive overall.

## Methods

### Participants

The data for this study came from the Psychology and Behavior Investigation of Chinese Residents database. This study was carried out across an extensive geographical expanse within China, encompassing 148 cities; 202 districts; 390 townships, towns, and streets; as well as 780 communities and villages, between June 20 and August 31, 2022. The sampling strategy used for this survey was multistage, meticulously informed by quota attributes derived from China’s seventh national census data at the city level. These attributes included sex, age, and urban-rural distribution, ensuring a representative and stratified selection of participants. The detailed application of the quota method has been thoroughly documented in a preceding investigation by Wang et al [22]. This study was registered in the China Clinical Trial Registry (ChiCTR2200061046).

The study used the web-based Questionnaire Star platform for questionnaire distribution. Participants were eligible if they were Chinese nationals aged ≥12 years, voluntarily participating, comprehending each questionnaire item, and completing the questionnaire independently. For those with limited mobility but intact cognitive function, investigators conducted one-on-one interviews, offering necessary assistance without influencing responses, thereby ensuring inclusivity and data integrity.

### Ethical Considerations

This study was approved by the Ethics Research Committee of the Health Culture Research Center of Shaanxi (JKWH-2022-02). Informed consent was obtained from all participants. All data were collected anonymously and kept confidential. Participants did not receive any material or monetary rewards for participation. We ensure that all the images in the written materials published by this study do not contain the identity information of individual participants.

### Self-Administered Questionnaires

The self-administered portion of the questionnaire investigated the demographic characteristics of the participants. The demographic characteristics included sex (male and female), age (60-74 years and ≥75 years), income (≤Y 4000 and ≥Y 4000 [US $550]), place of residence (eastern region, central region, and western region), education level (senior high school and below, junior college and bachelor degree, and postgraduate degree and above), occupational status (employed, retired, and unemployed: unemployed, jobless, and no regular occupation), and health care cost-bearing method (purely health insurance, purely commercial insurance, mixed insurance, and no health insurance), and chronic disease status (no chronic disease and chronic disease). The dependent variable measured was willingness to use mHealth devices (rated on a scale from a score of 0=not accepted to 100=very accepted).

### Standard Scale

#### Health Literacy Questionnaire

In 2013, Sørensen et al [23] developed the 47-item European Health Literacy Survey Questionnaire, and in 2019, Duong et al [24] simplified the European Health Literacy Survey Questionnaire, creating a 12-item scale known as the Health Literacy Scale–short form (HLS-SF; HLS-SF12), designed to assess public health literacy in Asian countries. In 2023, Sun et al [25] sinicized HLS-SF12 using translation, reverse translation, and cultural debugging procedures. In 2023, Sun et al [26] applied the Mokken model in item response theory, classical test theory, to simplify HLS-SF12 (simplified to HLS-SF9). In this study, namely, the HLS-SF9 was used to measure the respondents’ health literacy. The scale consists of 3 dimensions—health care, disease prevention, and health promotion—with 9 entries, each of which is rated on a 4-point scale (1=very difficult, 2=difficult, 3=easy, and 4=very easy), and the standardized health literacy index (ie, the total score of the scale) was calculated using a formula, which ranges from 0 to 50, with the higher the index representing the higher level of health literacy. The formula was calculated as index = (mean – 1) × (50/3), where mean is the average of the scores of all the entries for each individual, 1 is the smallest possible value of the mean (at which point the minimum value of the index is 0), 3 is the range of the mean, and 50 is the maximum value of the index. The higher the index, the higher the level of health literacy of the respondent. The Cronbach α coefficient of the scale was 0.923.

#### The New General Self-Efficacy Scale

The New General Self-Efficacy Scale (NGSES) was developed by Chen et al [27] on the basis of the General Self-Efficacy Scale, developed in 2001. The members of the project team of the “2022 Survey of Psychology and Behavior of the Chinese Population” applied the Mokken model of item response theory and classical test theory to simplify the NGSES-8, reducing the original 8 items to 3 items, and forming a new general self-efficacy scale. The NGSES-8 was simplified from the original 8 items to 3 items to form the NGSES (Wu et al [28]). In this study, respondents’ self-efficacy was measured by the NGSES-3, which contains 3 entries on a 5-point Likert scale (1=strongly disagree and 5=strongly agree; the total scale score is the sum of the 3 entries, and the range of scale scores is from 3 to 15, with higher scores indicating higher levels of self-efficacy among respondents). It includes 3 entries on whether they can accomplish difficult tasks, whether they can successfully overcome many challenges, and whether they have the confidence to accomplish many different tasks effectively. The Cronbach α coefficient of the scale was 0.912.

#### The Perceived Social Support Scale

The Perceived Social Support Scale (PSSS) is used to assess participants’ perceptions of social support (Li et al [29]). The PSSS consisted of 3 parsimonious items in this study, assessing perceived emotional support from friends, family, and significant others. It has been validated to be well correlated with the original items’ scale, and the factor structure, reliability, and validity have also been well established (Wu et al [28]). Each item is scored on a 7-point scale of 1 to 7 (1=“strongly disagree” and 7=“strongly agree”). The summed scores on the PSSS range from 3 to 21 points, with higher scores representing greater perceived social support. The Cronbach α coefficient of the scale was 0.902.

#### The Family Communication Scale

The Family Communication Scale-10 (FCS-10) was used to evaluate the respondents’ family communication. The scale was developed by Olson and Barnes and was sinicized by Guo N et al (Kwon et al [30] and Guo et al [31]). The FCS-10 consists of 10 entries on a 5-point Likert scale (1=strongly disagree and 5=strongly agree; the total scale score is the sum of the 10 entries, and the scale score ranges from 10 to 50, with higher scores indicating that the respondent’s family communication is better). The purpose of the scale is to measure the quality of communication among family members with regard to the exchange of ideas, information, level of concern, openness, confidence, and emotions among family members. The Cronbach α coefficient of the scale was 0.957.

#### The Health Effect Values Visual Analog Score

The Health Effect Values Visual Analog Score (EQ-5D-VAS) is part of the European Five-Dimensional Health Scale. In this study, respondents rated the goodness of their health using the EQ-5D-VAS. The scale is a numerical range from 0 to 100, where 100 represents the respondent’s perceived best possible health condition and 0 represents the worst. Respondents are asked to indicate their self-assessed health status by selecting a whole number between 0 and 100.

#### The Identity Bubble Reinforcement Scale

The Identity Bubble Reinforcement Scale focuses on the social and information homogenization tendency brought about by identity-driven social media use from a psychosocial point of view. It is mainly used to measure the tendency of social media users to get involved in identity-driven online bubbles (bubble refers to homogenized information environments) and to emphasize the impact of identity-driven social activities on individual behavior within social media. The scale consists of 9 entries divided into 3 dimensions—social identity (3 entries), homogenization (3 entries), and information bias (3 entries)—and is based on a 10-point scale ranging from 1 (not at all like me) to 10 (not at all like me), with higher scores representing a more significant tendency to homogenize an individual’s identity-driven social media activities (Kaakinen et al [32]). The Cronbach α coefficient of the scale was 0.948.

### Data Exclusion

**Figure 2:**
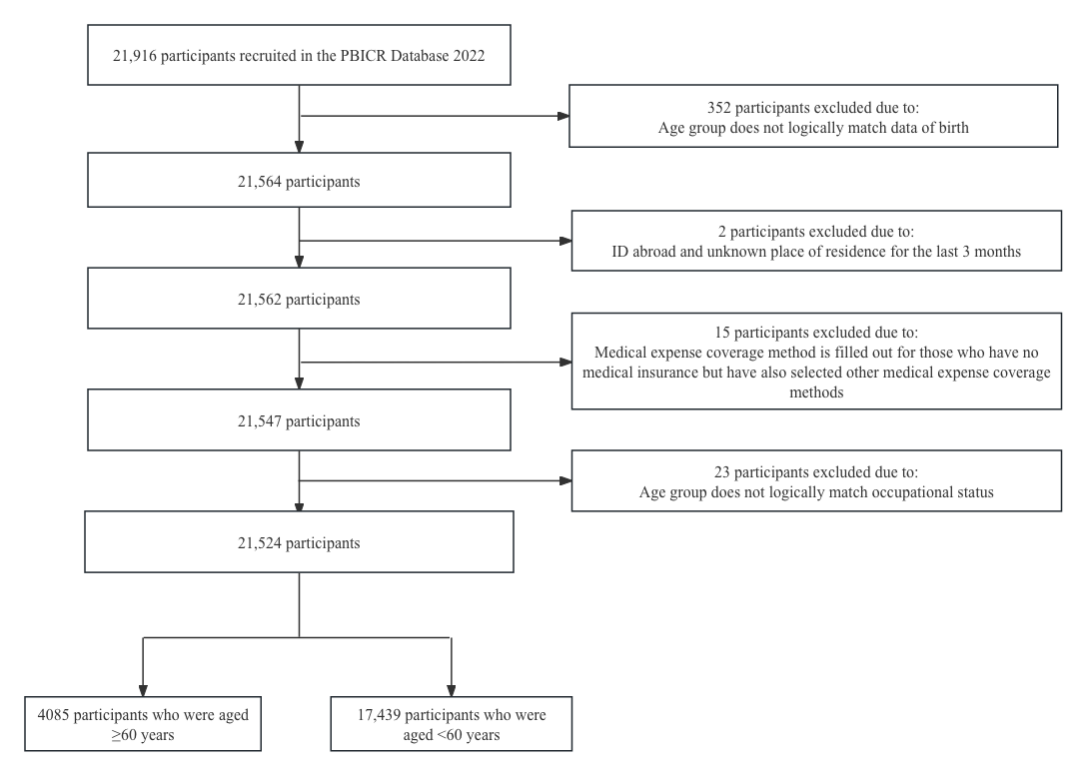
Flowchart of sample selection of older adults’ willingness to use mobile health devices. PBICR: Psychology and Behavior Investigation of Chinese Residents.

The specific data selection process is shown in Figure 2. The total sample size of the Psychology and Behavior Investigation of Chinese Residents database in 2022 was 21,916, of which 2 participants’ ID abroad and unknown place of residence for the last 3 months, and 390 participants filled in information that was illogical. Thus, 392 participants were excluded, and 21,524 participants were retained. Among these 21,524 participants, 17,439 (81.02%) were aged <60 years and 4085 (18.97%) were aged ≥60 years.

### Statistical Analysis

Data analysis was conducted using SPSS (version 26.0; IBM Corp) and RStudio (RStudio, Inc), and all comparisons were 2-tailed, with *P* values <.05 considered statistically significant. The basic information of participants, along with their scores on the quality of life scale, family communication scale, health literacy scale, and willingness to use mHealth devices, were first analyzed descriptively. This was followed by univariate analysis of each potential explanatory variable: binary independent variables were analyzed using a 2-tailed *t* test, multicategorical independent variables with an *F* test, and continuous independent variables with correlation analysis. On the basis of these results, a model combining multiple stepwise linear regression and classification and regression tree (CART) decision tree models was developed to explore the factors influencing older adults’ willingness to use mHealth devices.

## Results

### Characteristics of the Participants

A total of 21,916 samples were collected in this study, and 392 (1.78%) sets of abnormal data were excluded by logical checking, while 21,524 (98.21%) sets of valid data were retained. The overall sample consisted of 18.98% (n=4085) older adult respondents (aged ≥60 years) and 81.02% (n=17,439) non–older adult respondents (aged <60 years). The mean score of all respondents’ willingness to use mHealth devices was 68.14 (SD 25.30), with non–older adult respondents (69.18, SD 25.23) having a higher mean score of willingness to use mHealth devices than older adult respondents (63.70, SD 25.11).

There were 49.91% (n=2039) male participants and 90.8% (n=3709) Han Chinese in the sample of 4085 older adults, with a preponderance of younger older adults aged <75 years and >60 years (n=3383, 82.82%); most respondents’ per capita monthly income of the household were ≤Y 4000 (US $550; n=2560, 62.67%); most respondents had a senior high school and below education (n=3523, 86.24%); 55.25% (n=2257) of the respondents are retired; 41.42% (n=1692) of the respondents are unemployed; and only 3.33% (n=136) of the respondents are still employed. Some (n=1471, 36.01%) respondents have lived in the eastern region for the last 3 months, 47.69% (n=1948) of the respondents have lived in the western region for the last 3 months, and 16.3% (n=666) of the respondents have lived in the central region for the last 3 months.

### Each Scale Score of Participants

In total, 4085 respondents had a mean score of 69.86 (SD 18.55) for quality of life (EuroQol Visual Analog Scale), 29.74 (SD 9.85) for health literacy (HLS-SF9 scale), 35.40 (SD 12.05) for identity bubble (Identity Bubble Reinforcement Scale), 14.79 (SD 3.63) for perceived social support (PSSS), 10.51 (SD 2.42) for self-efficacy (NGSES), 4.44 (SD 1.54) for loneliness (T-ILS scale), and 36.92 (SD 7.63) for family communication (FCS-10 scale).

### Willingness to Use mHealth Devices Among Chinese Older Adults

The mean score of the older adult population’s willingness to use mHealth devices in this study was 63.70 (SD 25.11; median 65.00). To explore the distribution of older adult populations’ willingness to use mHealth devices, the continuous variable of willingness to use mHealth devices was divided into 10 groups (0-10, 11-20, ... 91-100), and the number of respondents in each willingness to use subgroup was counted and presented in the form of a frequency distribution histogram. The specific results are shown in Multimedia Appendix 1.

As shown in Multimedia Appendix 1, older adults’ willingness to use mHealth devices is roughly based on a clear dividing line of 40 points, with 17.36% (n=709) of the 4085 older adults having a willingness to use mHealth devices with scores <40 and 82.64% (n=3376) of older adults having a willingness to use mHealth devices with scores >40, with the largest number of older adults (n=664, 16.25%) having a willingness to use mHealth devices located in the range of 91 to 100. Subsequently, we observed 3 specific attitudinal older adult populations with respect to their willingness to use mHealth devices, namely, the strong nonwillingness to use mHealth devices population (scored 0) and the strong willingness to use mHealth devices population (scored 100). Some (n=51, 1.25%) older adults had no willingness to use mHealth devices at all, and 11.43% (n=467) of the older adults had a strong willingness to use mHealth devices.

### Univariate Analysis of the Willingness of Older Adult Population to Use mHealth Devices

Because the dependent variable in this study is a continuous variable, a *t* test was used for binary independent variables, an *F* test was used for multicategorical independent variables, and correlation analysis was used for continuous independent variables. Most of the study variables were associated with willingness to use mHealth devices (*P*<.001; Table 1).

**Table 1:** Descriptive statistics of the willingness to use mobile medical devices among the older adults (N=4085).

Variables			Older adults, n (%)	Mobile health devices willingness, mean (SD)	*t* test (*df*)		*F* test (*df*)		Spearman rank correlation coefficient		*P* value	
**Predisposing characteristics**												
	**Sex**					–1.02 (4083)		—^a^		—		.31
		Male	2039 (49.91)	64.10 (25.32)								
		Female	2046 (50.09)	63.29 (24.90)								
	**Ethnicity**					–1.90 (4083)		—		—		.06
		Han	3709 (90.80)	63.46 (25.21)								
		Minority	376 (9.20)	66.03 (24.02)								
	**Age group (years)**					2.67 (964.33)		—		—		.008
		60-74	3383 (82.82)	64.20 (24.90)								
		≥75	702 (17.18)	61.27 (26.79)								
	**Education level**					—		24.93 (2,4082)		—		<.001
		Senior high school and below	3523 (86.24)	62.72 (25.26)								
		Junior college and bachelor’s degree	504 (12.34)	68.64 (23.06)								
		Postgraduate degree and above	58 (1.42)	79.98 (23.05)								
	**Living region**					—		17.12 (2,4082)		—		<.001
		Eastern region	1471 (36.01)	66.55 (23.08)								
		Central region	666 (16.30)	60.36 (27.52)								
		Western region	1948 (47.69)	62.68 (25.51)								
	**Occupational status**					—		14.67 (2,4082)		—		<.001
		Employed	136 (3.33)	64.55 (28.11)								
		Retired	2257 (55.25)	65.5 (324.06)								
		Unemployed	1692 (41.42)	61.18 (26.01)								
	**Social status**					—		45.59 (2,4082)		—		<.001
		Low	180 (4.41)	57.05 (31.20)								
		Medium	3099 (75.86)	62.22 (24.58)								
		High	806 (19.73)	70.87 (24.25)								
	**Health literacy**					–11.36 (3902.26)		—		—		<.001
		Low	2315 (56.67)	59.88 (25.30)								
		High	1770 (43.33)	68.69 (23.97)								
	**Self-efficacy**					–9.97 (3757.46)		—		—		<.001
		Low	2269 (55.54)	60.21 (23.99)								
		High	1816 (44.46)	68.06 (25.79)								
	**Whether children living together**					—		2.14 (2,4082)		—		.12
		Yes	1786 (43.72)	64.55 (24.69)								
		No	1525 (37.33)	62.74 (24.95)								
		Not filled	774 (18.95)	63.60 (26.33)								
**Enabling resources**												
	**Per capita monthly income of the household (US $)**					–6.29 (3575.12)		—		—		<.001
		≤Y 4000 (US $550)	2560 (62.67)	61.87 (26.29)								
		>Y 4000 (US $550)	1525 (37.33)	66.77 (22.68)								
	**How medical expenses are borne**					—		5.54 (3,4081)		—		<.001
		Only medical health insurance	3175 (77.72)	62.87 (25.15)								
		Only commercial health insurance	91 (2.23)	66.63 (19.47)								
		Hybrid insurance	587 (14.37)	66.03 (24.60)								
		None	232 (5.68)	68.00 (27.01)								
	**Perceived social support**					—		63.09 (2,4082)		—		<.001
		Low	338 (8.27)	58.79 (21.53)								
		Middle	2025 (49.57)	60.20 (24.30)								
		High	1722 (42.15)	68.77 (25.81)								
	**Family health**					—		12.45 (2,4082)		—		<.001
		Poor	1237 (30.28)	61.18 (22.81)								
		Moderate	1471 (36.01)	63.59 (26.07)								
		Excellent	1377 (33.71)	66.07 (25.82)								
	**Quality of life**					–14.49 (4083)		—		—		<.001
		Low	2864 (70.11)	60.07 (24.14)								
		High	1221 (29.89)	72.20 (25.31)								
	Family communication, mean (SD)		—	—	—		—		0.142		<.001	
	Identity bubbles, mean (SD)		—	—	—		—		0.179		<.001	
**Need**												
	**Chronic diseases conditions**					—		22.84 (2,4082)		—		<.001
		Having chronic diseases related to mobile health devices	1682 (41.18)	62.09 (25.66)								
		Having chronic diseases unrelated to mobile health devices	672 (16.45)	60.07 (24.37)								
		Not having chronic diseases	1731 (42.37)	66.67 (24.54)								
	**Loneliness**					3.08 (4083)		—		—		.002
		Not alone	2832 (69.33)	64.50 (24.76)								
		Alone	1253 (30.67)	61.88 (25.80)								
	**Depression**					—		17.06 (4,4080)		—		<.001
		No	1894 (46.36)	66.92 (25.31)								
		Mild	1363 (33.37)	60.32 (24.53)								
		Moderate	559 (13.68)	61.46 (24.17)								
		Moderate to severe	218 (5.34)	60.91 (26.03)								
		Severe	51 (1.25)	70.90 (23.81)								
	**Anxiety**					—		10.18 (3,4081)		—		<.001
		No	2282 (55.86)	65.48 (25.81)								
		Mild	1324 (32.41)	60.96 (23.77)								
		Moderate	414 (10.13)	62.07 (23.88)								
		Severe	65 (1.59)	67.31 (28.99)								

^a^Not available.

### What Are the Factors Influencing the Willingness to Use mHealth Devices Among Chinese Older Adults?

#### Factors Influencing the Willingness to Use mHealth Devices Among Chinese Older Adults Based on Multiple Stepwise Linear Regression

This study used multiple stepwise linear regression to explore the factors affecting the willingness of older adults to use mHealth devices, and the specific results of multiple stepwise linear regression are shown in Table 2. The 13 main factors influencing older adults’ willingness to use mHealth devices were education level, occupational status, social status, health literacy, self-efficacy, whether children were living together, per capita monthly income of the household, perceived social support, quality of life, family communication, identity bubbles, chronic disease conditions, and depression. In terms of predisposing characteristics, older adults who lived with their children (*P*=.02; β=.034), had high health literacy (*P*<.001; β=.089), had high self-efficacy (*P*=.02; β=.043), had high social status (*P*<.001; β=.085), were unemployed (*P*=.02; β=.037), and had the highest education level of postgraduate degree and above (*P*=.007; β=.040) were willing to use mHealth devices. In terms of enabling resources, older adults with a household per capita monthly income of ≥Y 4000 (*P*=.002; β=.048), a high quality of life (*P*<.001; β=.149), high perceived social support (*P*<.001; β=.096), and a lower family communication level (*P*<.001; β=–.071) were more willing to use mHealth devices. In terms of need, older adults who did not have a chronic disease (*P*=.001; β=.049) and had mild depression (*P*=.007; β=.040) were less willing to use mHealth devices.

**Table 2:** Multiple stepwise linear regression results of the older adults’ willingness to use mobile health devices.

Variables					Unstandardized coefficients, B (SE)			Standardized coefficients, β (95% CI)			*t* test			*P* value			Variance inflation factor
**Predisposing characteristics**																	
	**Education level (reference: senior high school and below)**																
		Postgraduate degree and above	8.480 (3.165)			0.040 (2.276 to 14.684)			2.680			.007			1.018		
	**Occupational status (reference: employed)**																
		Unemployed	1.846 (0.784)			0.037 (0.308 to 3.384)			2.353			.02			1.105		
	**Social status (reference: low)**																
		High	5.370 (0.954)			0.085 (3.500 to 7.241)			5.628			<.001			1.047		
	**Health literacy (reference: low)**																
		High	4.533 (0.879)			0.089 (2.810 to 6.255)			5.160			<.001			1.376		
	**Self-efficacy (reference: low)**																
		High	2.155 (0.932)			0.043 (0.327 to 3.984)			2.312			.02			1.559		
	**Whether children living together (reference: no)**																
		Yes	1.741 (0.752)			0.034 (0.266 to 3.216)			2.314			.02			1.012		
**Enabling resources**																	
	**Per capita monthly income of the household (reference: ≤Y 4000** **(US $550)**																
		>Y 4000 (US $550)	2.491 (0.819)			0.048 (0.885 to 4.097)			3.040			.002			1.140		
	**Perceived social support (reference: low)**																
		High	4.882 (0.946)			0.096 (3.028 to 6.736)			5.163			<.001			1.583		
	**Quality of life (reference: low)**																
		High	8.197 (0.864)			0.149 (6.504 to 9.890)			9.491			<.001			1.135		
	Family communication			–0.234 (0.065)			–0.071 (–0.361 to –0.108)			–3.630			<.001			1.759	
	Identity bubbles			0.178 (0.033)			0.085 (0.113 to 0.243)			5.339			<.001			1.170	
**Need**																	
	**Chronic diseases conditions (reference: having chronic diseases related to mobile health devices)**																
		Not having chronic diseases	2.513 (0.770)			0.049 (1.004 to 4.022)			3.264			.001			1.051		
	**Depression (reference: no)**																
		Mild	–1.860 (0.818)			–0.035 (–3.463 to –0.256)			–2.274			.02			1.080		

#### Factors Influencing the Willingness to Use mHealth Devices Among Chinese Older Adults Based on CART Decision Tree Model

**Figure 3:**
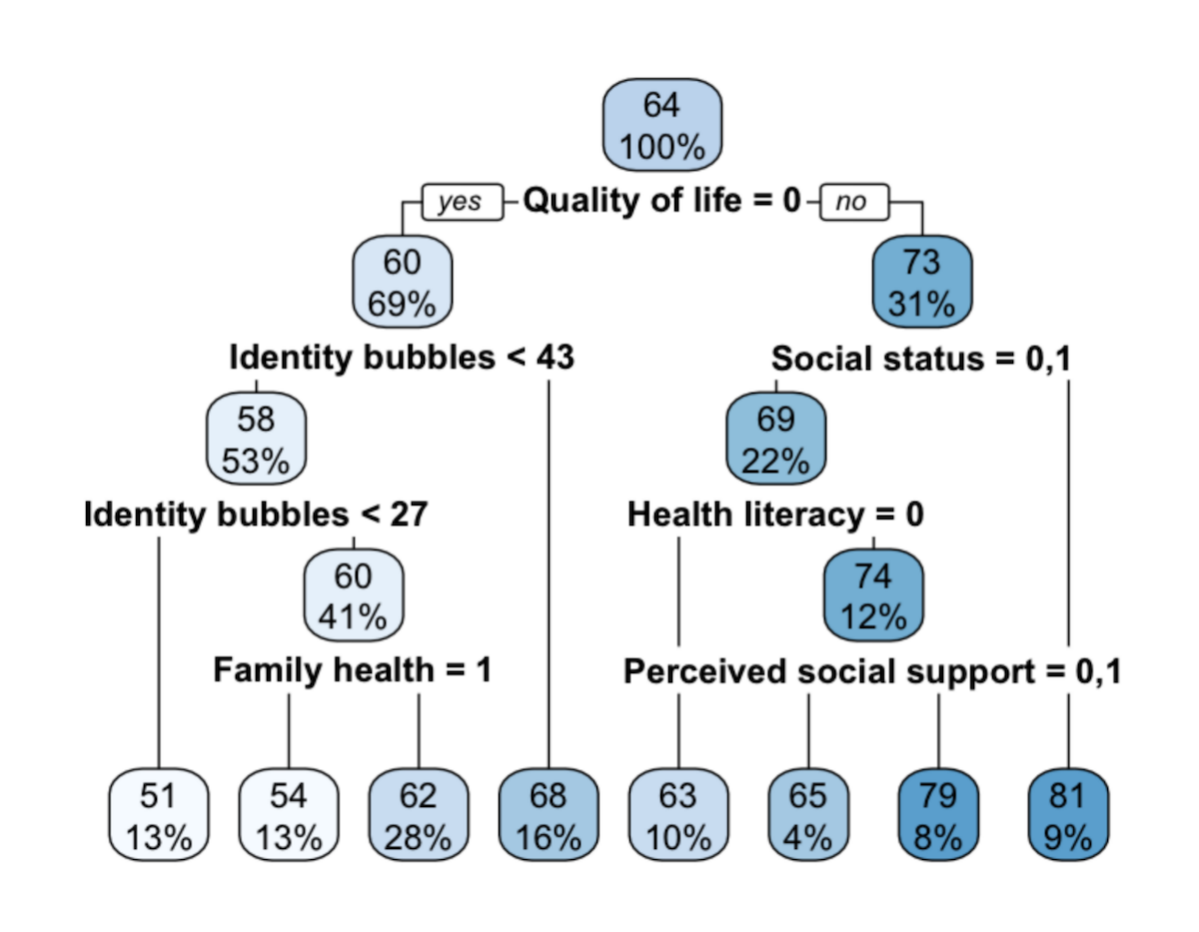
Classification and regression tree (CART) decision tree results of older adults’ mobile health device use willingness.

Multiple stepwise linear regression can explore the main influencing factors of the use of mHealth devices for the older adults, but it is not possible to intuitively understand the relative importance of the influencing factors and the interaction between the influencing factors; therefore, this study uses the CART decision tree model to visualize the influencing factors (with a training set:test set ratio of 70%:30%) to explore the interaction between the influencing factors. The specific results are shown in Figure 3. Figure 3 shows that quality of life is the most important factor influencing the willingness to use mHealth devices among older adults, and the willingness to use mHealth devices among older adults with high quality of life (73 points) is significantly higher than that of older adults with low quality of life (60 points). For the older adult group with low quality of life, identity bubbles is the main influencing factor on their willingness to use mHealth devices. When the identity bubbles score is ≥43, the mean value of the older adults’ willingness to use mHealth devices is 68 points, and when the identity bubbles score is <43, the mean value of the older adults’ willingness to use mHealth devices is 58 points. And then if the identity bubbles score is <27, the mean value of willingness to use mHealth devices for such older adults is 51 points, and if the identity bubbles score is ≥27, family health becomes the main factor influencing older adults’ willingness to use mHealth devices, with the mean value of willingness to use mHealth devices for the older adults with moderate family health being 54 points and the mean value of willingness to use mHealth devices for the older adults with poor family health or excellent family health being 62 points. For older adults with high quality of life, social status is the main factor influencing older adults’ willingness to use mHealth devices, with the mean value of mHealth devices for older adults with high social status being 81 points, while older adults with medium and low social status need to further judge their health literacy, with the mean value of mHealth devices for older adults with low health literacy being 63 points, and older adults with high health literacy needed to be further judged in terms of their sense of perceived social support, with older adults with high perceived social support (79 points) having a significantly higher willingness to use mHealth devices than older adults with low perceived social support (65 points). This shows that older adults with high quality of life and high social status have the highest willingness to use mHealth devices (81 points), while older adults with low quality of life and low status bubbles (<27 points) have the lowest willingness to use mHealth devices (51 points).

## Discussion

### Overview

This study found that Chinese older adults have positive attitudes toward using mHealth devices, with significant positive effects of education, quality of life, self-efficacy, and health literacy on the use of mHealth devices at the individual level; a positive effect of perceived family social status at the social level; and a negative effect of family communication on their willingness to use the devices. Social-level perceived social support and identity bubble had a significant positive effect on using mHealth devices.

### Principal Findings

The study finds that the gap in willingness to use mHealth devices between older adults and non–older adults is relatively small. The average willingness score for older adults is 63.70, compared to 69.18 for non–older adult respondents. This differs from the conclusions of existing studies, which indicate that older adults have a significantly lower proportion of using electronic health devices to access information compared to non–older adult groups [33,34]. A Canadian-based study also showed significant differences in the use of smart devices between older and younger adults, with nearly half of them using smartphones but not downloading health-related apps [35]. This may be partly because older adults have greater health needs [36]. Meanwhile, it may also be attributed to the fact that today’s digital technologies have increasingly prioritized age-friendly design, making older adult–friendly devices more accessible and ensuring that older adults are no longer left on the periphery of digitalization [37]. Studies have shown that, most of the time, older and younger adults share similar preferences for using electronic health devices. When the devices are impractical or lack inclusive design, people across different groups tend to reduce their use [38].

In terms of research methodology, this study uses a combined model of logistic regression and decision tree analysis to comprehensively and rigorously examine the factors influencing older adults’ willingness to use mHealth devices. Both methods identify key determinants, such as quality of life, identity bubble, perceived social support, self-efficacy, family communication, per capita monthly household income, and health literacy. The logistic regression model further highlights additional demographic factors, including region of residence, education level, and chronic disease status. In contrast, the decision tree model emphasizes the stronger impact of subjective psychological factors.

### Factors Influencing Older Adults’ Willingness to Use Health Devices

This study focuses extensively on the adaptability of the older adult population in the context of digital health and smart health care. Using older adults’ use of mHealth devices as a starting point, it explores their willingness to use such devices and the influencing factors. Using a comprehensive systematic framework of predisposing characteristics, enabling resources, and needs, this study conducts a thorough analysis of variables and fits the data using binary logistic regression. The findings indicate that educational level, perceived family social status, perceived social support, self-efficacy, quality of life, health literacy, identity bubble, and intrafamily communication significantly influence the older adults’ willingness to use mHealth devices. This not only validates the Behavioral Model of Health Services Use in explaining the use of health care services among the older adults but also demonstrates its applicability in the digital age.

In terms of predisposing factors, this study reveals that perceived family social status, educational level, and health literacy are positively correlated with older adults’ willingness to use mHealth devices. This conclusion complements existing research, which often focuses on the relationship between objective social class and status and the use of digital devices. For example, some studies suggest that individuals with higher social class are better equipped to use digital devices in the field of education [39]. However, there has been relatively little research on the relationship between subjective class perception and the use of digital devices. This study creatively establishes a link between the 2 and focuses specifically on the health domain. In terms of health literacy, research shows that the application of digital health technologies is highly correlated with users’ health literacy [40]. In terms of education and health literacy, this study aligns with the conclusions of existing research. Research showed that digital health education can enhance people’s understanding of diseases and is more effective than traditional education methods in improving overall health [41]. Therefore, older adults who have received digital health education and possess higher health literacy are more motivated to use digital health devices.

This finding can be elucidated through the lens of the digital divide. Concerning perceived family social status, on one hand, the formation of the digital divide often relates to the objective social strata within families, encompassing dimensions such as income [42] and sociocultural factors. Individuals with higher incomes are more likely to have greater access to the internet and IT [43]. Moreover, formal and informal rules regarding inequality within social cultures also influence the digital divide experienced by different groups [44]. On the other hand, as a subjective variable, perceived social class is influenced by objective social strata and often correlates more closely with individuals’ life and psychological states [45]. Therefore, individuals who perceive themselves as belonging to a higher social class often possess the capability to access digital devices, use digital technologies effectively and reasonably, and fulfill their personal needs through these technologies. They also exhibit higher levels of acceptance and inclusiveness toward digital technologies, resulting in a greater willingness to use mHealth devices. It is important to note that existing research typically describes the phenomenon of the digital divide among the older adults from the perspective of objective social strata or social capital, without extensively exploring the impact of subjective social class perceptions on older adults’ preferences for using mHealth devices. Thus, this study contributes by supplementing existing research from a subjective psychological perspective.

Research on the digital divide and digital engagement among older adults often presents 2 perspectives concerning the variables of education level and health literacy. One perspective considers the integration or participation of older adults in digital life because of the 3-level digital divide. It suggests that individuals with higher education levels and greater technological understanding are generally more active in using digital devices. Accordingly, older adults benefit from mastering digital technologies and actively engaging in digital life [46]. The second perspective views “digital disengagement” among older adults as a fourth type of digital divide [47]. It contends that even as older adults enhance their digital literacy and acquire basic knowledge and skills in IT while integrating into the digital society, they may become unwilling to use digital technologies due to discriminatory content and other factors present in digital applications aimed at older demographics [48]. The results of this study support the first perspective, indicating that overall, individuals on the periphery of the digital society within the older adult population exhibit relatively lower willingness to use digital devices. This finding aligns with some existing research in this field. A survey of the Polish population reveals that the educational level of older adults and their motivation to use digital devices are significant variables influencing their level of digital participation [49]. There is also evidence suggesting that through professional training or digital literacy education, older adults’ digital skills and literacy can significantly improve [50]. These digital skills form the foundation for older adults to use digital devices [22]. Therefore, reducing the digital divide and increasing familiarity with digital devices among older adults may be a way to enhance their willingness to use such devices.

Furthermore, this study found that self-efficacy significantly influences older adults’ use of mHealth devices, consistent with previous research findings. The research indicates a correlation between willingness to use digital technology and self-efficacy among retired older adult populations [51]. On one hand, learning to use digital technology can enhance self-efficacy among older adults [52]; on the other hand, lower self-efficacy can diminish their ability to use smart devices [53]. These findings provide a basis for the conclusions drawn in this study.

In terms of facilitating resources, first, this study found a significant positive correlation between perceived social support, identity bubbles, and the willingness of older adults to use mHealth devices. This aligns with previous research findings. Scholars argue that perceived social support is among the crucial factors influencing tablet device use among older adults [54]. This can be explained using social identity theory. Research indicates that there are often stereotypes about older adults within society, and younger individuals who have more contact with older adults tend to possess greater knowledge about aging-related issues. They also show more understanding and fewer biases toward older adults [55]. Due to their greater proficiency and flexibility in using mHealth devices, younger individuals often serve as exemplary models for older adults when they experience higher levels of social acceptance and reduced stereotyping. Consequently, older adults are more likely to learn from younger generations in using mHealth devices and receive greater encouragement in digital device use. Therefore, increased social support and a sense of belonging on social media platforms among older adults enhance their willingness to engage with mHealth devices. Furthermore, the findings of this study validate hypotheses derived from social capital theory, which posits that objective resources and subjective identities and trust within social relationships positively influence internet use [56]. Therefore, older adults’ identification with internet communities and their level of digital identity bubbles also positively influence their willingness to use digital devices.

Second, this study found that family communication significantly negatively impacts the willingness of older adults to use mHealth devices, indicating that greater family communication correlates with a reduced inclination among older adults to use such devices. This finding contrasts with existing research conclusions. Existing studies suggest that intergenerational family networks are crucial in enhancing older adults’ digital literacy and can increase their willingness to use relevant digital devices [57]. This could be due to several factors. On one hand, there are distinctions between health devices and typical digital social devices. On the other hand, older adults experience significant social isolation, particularly those living alone and lacking care from their children, thereby increasing their willingness to use digital devices [58]. The application of digital devices by older adults often serves as a substitute or supplement to real-world social and health needs. When these needs are adequately met in reality, there is a decrease in both the willingness and use rates of relevant digital devices among older adults [59], which could serve as an explanatory mechanism for the conclusions drawn in this study.

In terms of demand, this study found that quality of life has a significantly positive impact on older adults’ willingness to use mHealth devices. Existing research has laid the groundwork for this conclusion. Studies, such as those involving telephone interviews with older adults in Hungary during COVID-19, have identified substantial demand for digital health solutions among older adult populations, highlighting its importance as a critical application area [37]. This suggests that older adults’ subjective perception of health and their objective life circumstances both influence their demand for and willingness to use digital health devices. This supports the conclusions drawn in this study.

It is noteworthy that explanations of these 3 dimensions are not entirely distinct; in fact, these variables collectively exert a systematic and comprehensive influence on older adults’ willingness to use mHealth devices.

### Future Prospects

This study has the following strengths: regarding the research topic, it explores the psychological foundation of the digital divide among older adults from the perspective of subjective willingness, enriching the structural discussion as corroborated by existing research. In terms of data, the study uses scientifically sampled large-scale samples of older adults, encompassing rich variables and comprehensive discussions, thereby supplementing existing research with empirical analysis from China. Theoretically, the study systematically categorizes variables and discusses the theoretical relationship between subjective class perception and the digital divide, while validating social identity and social capital theories, contributing to theoretical significance.

However, the limitations of this study primarily stem from its cross-sectional design, which offers only a preliminary exploration of influencing factors without addressing potential mechanisms or causal effects. In addition, the lack of comprehensive external databases for triangulating results in the decision tree analysis is another limitation. Furthermore, the study lacks subgroup explanations. In reality, older adults with different characteristics may not score similarly on various influencing factors, and their motivations for using mHealth devices can vary in terms of initiative, passivity, and purpose. In addition, the study relies on a single-item measure for the dependent variable—older adults’ willingness to use mHealth devices. While a single-item measure offers simplicity and ease of administration, it may lack the depth and reliability of multiitem scales, which could better capture the complexity and nuances of willingness and actual behaviors. This limitation might lead to measurement bias and restrict the robustness of the findings.

To further delve into this topic in the future, we propose the following questions for in-depth discussion: Do older adults use medical devices differently than the general population? first, this study found that there was no significant difference in the willingness of older adults to use health devices compared to the general population, which contrasts with previous researches. Existing studies indicate that younger age groups prefer generative artificial intelligence that enhances productivity compared to generative artificial intelligence primarily used in health settings. In addition, research suggests that the internet has a greater impact on younger individuals than on older adults. However, individuals aged ≥60 years tend to approach the use of IT devices with caution, shyness, and deliberation [60]. It suggests a need for further exploration into the underlying reasons for this distinction.

Second, do the influencing factors on older adults’ willingness to use health devices similarly apply to different age groups? Research indicates that for children and adolescents, family factors and parent-child relationships often have a significant positive impact on their use of digital devices [61], yet there remains a lack of research on their use of health devices. Therefore, this could serve as a direction for further exploration in the future.

### Conclusions

The results of this study showed that Chinese older adults’ willingness to use mHealth devices was positive overall. The positive state of family communication, on the contrary, reduced their willingness to use. This suggests that particular attention should be paid to the singleton and widowed older adult groups, where the lack of family communication may translate into higher acceptance and dependence on mHealth devices. Therefore, policy development should focus on providing free or subsidized health devices for these groups, and in addition, when designing promotional strategies, attention should be paid to balancing the relationship between family communication and technology acceptance, and at the same time, strengthening family digital health education to promote the incorporation of technology in ways that can enhance rather than weaken family health management and emotional connection, help fill the gaps in older adult care services and improve health management, and realize the harmonious coexistence of technology and humanistic care.

## Appendix

Multimedia Appendix 1 Frequency distribution histogram results of the older adults’ willingness to use mobile health devices.

## Abbreviations

CART: classification and regression tree
FCS-10: Family Communication Scale-10
HLS-SF: Health Literacy Scale–short form
NGSES: New General Self-Efficacy Scale
PSSS: Perceived Social Support Scale
