# Supplementary figures and images for: Analysis of Factors Influencing the Willingness of Chinese Older Adults to Use mHealth Devices: Nationwide Cross-Sectional Survey Study
Source: J Med Internet Res. 2025 Mar 4;27:e66804. doi: 10.2196/66804 (PMC11920664; doi:10.2196/66804)

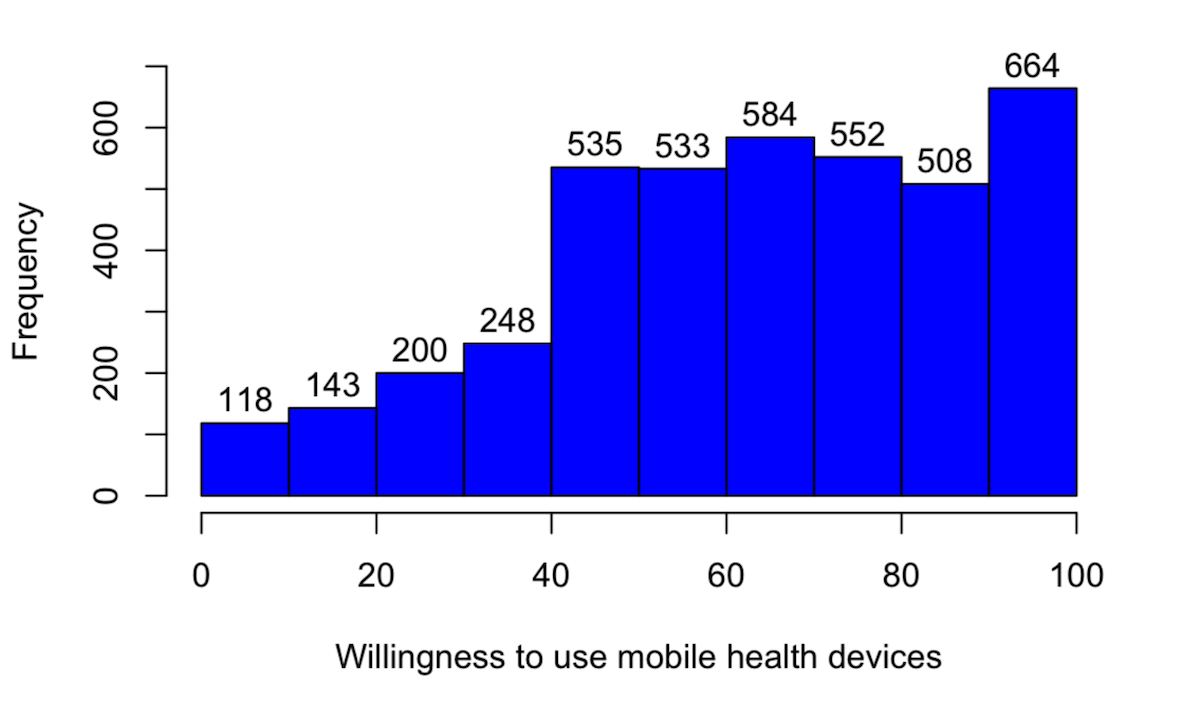

Supplement: Multimedia Appendix 1 [file jmir_v27i1e66804_app1.png]
